# Pennsylvania State Board of Veterinary Medical Examiners

**Published:** 1900-03

**Authors:** W. Horace Hoskins

**Affiliations:** Secretary


Secretary.

### PENNSYLVANIA STATE BOARD OF VETERINARY MEDICAL EXAMINERS.

The midwinter examinations of this board were held in Philadelphia on December 18 and 19, 1899. The following applicants for examination presented themselves: Dr. William J. Waugh, graduate of the Ontario Veterinary College, late veterinarian of the United States Army, located at Washington, Pa., and Dr. Warren J. Fretz, graduate of the American Veterinary College, located at Perkasie, Pa.

The Secretary reported a number of complaints alleging violations of the State laws, most of which had been adjusted without the necessity of recourse to law. In the case of one or two alleged veterinary dentists vigorous prosecution was authorized.

W. Horace Hoskins,
Secretary.

